# Buffalo Academy of Medicine

**Published:** 1898-08

**Authors:** Thomas F. Dwyer


### Society Proceedings.

### BUFFALO ACADEMY OF MEDICINE.

REPORTED BY THOMAS F. DWYER, M. D., Secretary.

*Annual meeting held at the Academy Parlors, Palace Arcade, Tuesday evening, June 14, 1898.*

THE president, Dr. LUCIEN HOWE, called the meeting to order at 9 p. m. The minutes of the last annual meeting were read and approved. The secretary of the academy and council then presented the following report which, on motion of Dr. W. C. KRAUSS was received and ordered spread on the minutes.

### ANNUAL REPORT OF THE SECRETARY.

*Gentlemen*—Twenty-nine applications for membership have been received and elected members during the last fiscal year, of which twenty-two were resident fellows and seven non-resident fellows. This is the largest increase in membership in any one year since the organisation of the academy. One resident fellow has been transferred to nonresident fellowship and one nonresident has been returned to resident fellowship. One resignation has been received and accepted during the year. The academy has lost by death two honored members, Dr. William S. Tremaine and Dr. John Cronyn. An amendment to Article III. of the by-laws was offered and favorably acted upon, changing the time of election between the hours 4 and 6 p. m. to 8 and 9 p. m. on the day of the annual meeting in June. During the summer a program for the session of 1897-98 was arranged by the council and a copy was sent to the members of the academy and to the profession of Buffalo. This plan having been so successful, the council recommends to the academy the advisability of continuing the formation and issuing of programs for the entire session.

One special meeting was called on March 12, 1898, to determine what action, if any, shall be taken concerning, first, proposed legislation in regard to Hamburg canal; second, the Brush-Davis telephone bill. On the former a committee of three was appointed and reported lengthy resolutions favoring the abatement of this menace to public health, which were unanimously adopted and copies sent to the members of the legislature from Erie county. The Brush-Davis telephone bill was decided to be foreign to the purposes of the society and the proposition to consider was laid on the table. Four regular meetings were held and entertained in turn by the sections of the academy:

Section on obstetrics, June 22, 1897, Dysmenorrhea, Dr. M. D. Mann; section on medicine, September 13, 1897, Medico-legal aspects of cases of poisoning, with especial reference to restriction of sale of poisons, Dr. A. L. Benedict, Hon. Arthur W. Hickman; section on surgery, January 11, 1898, The parasitic origin of cancer, with demonstrations of the organism and microscopical specimens, Dr. Roswell Park; section on pathology, March 15, 1898, The anatomy and mechanism of the foot considered with reference to certain abnormal conditions, illustrated by stereopticon, Dr. L. A. Weigel, Rochester, N. Y.

The meetings of the academy have been very well attended during the past year. At the June meeting 23 were present, September meeting 52, January meeting 96, and the March meeting 31, an average attendance of 50½. The average time occupied by each meeting was 1 hour and 53 minutes. The council of the academy held seven meetings during the year, with an average attendance of 9 members.

THOMAS F. DWYER, *Secretary.*

The treasurer's annual report was read by the acting treasurer, Dr. Charles S. Jewett. Dr. Wm. G. Ring moved that the report be received and referred to an auditing committee. The secretary moved, as an amendment, that the auditing committee be also instructed to audit the accounts for the previous year as no report had been presented by the treasurer at the last annual meeting. The motion as amended was carried.

The president appointed Drs. F. E. L. Brecht and A. L. Benedict as the auditing committee.

Reports were received from the secretaries of the sections on medicine, surgery, obstetrics and pathology, and on motion were received and ordered placed on file.

Dr. A. G. Bennett, on behalf of the tellers, reported the result of the election as follows: President, Dr. Roswell Park; treasurer, Dr. Eugene A. Smith; trustee, Dr. B. G. Long.

The auditing committee reported having compared the report of the treasurer with the books and vouchers for the past two years and found them correct. Dr. De Lancey Rochester moved that the report be received and placed on file. Carried.

The retiring president, Dr. Lucien Howe, then read his annual address and in closing thanked the officers of the academy and its sections for their coöperation during the very successful year just past.

Dr. P. W. Van Peyma moved that the thanks of the academy be extended to the officers for their work of the past year. Carried.

The installation of officers being in order Dr. Roswell Park, the newly elected president, was escorted to the chair and introduced by the retiring president, Dr. Lucien Howe. Dr. Park said that he appreciated very deeply the compliments of this election and thanked the academy for the very great honor. In closing his remarks Dr. Park said that he thought some recognition of the academy proceedings should be taken by the local medical journal and hoped that some arrangements could be made to that end during the coming year.

Dr. W. C. Krauss moved that the president appoint a committee to take into consideration the publication of our transactions in the Buffalo Medical Journal and to report at the quarterly meeting, June 28, 1898. Carried.

Drs. W. C. Krauss and Lucien Howe were appointed as such committee.

Adjourned at 10.20 p. m.

---

QUARTERLY MEETING.

The quarterly meeting was held at the academy parlors, Palace Arcade, Tuesday, June 28, 1898. Meeting called to order at 8.45 p. m. by the president, Dr. Roswell Park. The minutes of the last quarterly meeting were read and approved. The council recommended the election of the following applicants: Drs. Marshal Clinton, H. R. Gaylord and William C. Barrett for resident membership and Dr. Charles A. Ellis, Sherman, N. Y., for non-resident membership. They were separately ballotted for and elected fellows of the academy. The resignation of the treasurer, Dr. Eugene A. Smith, who is at Camp Alger with the 65th Regiment, N. Y. Volunteers, was read and accepted. A motion that the thanks of the academy be tendered to Dr. Smith who had faithfully performed the duties of treasurer since the organisation was unanimously carried. Dr.

Charles S. Jewett was nominated and elected treasurer for the unexpired term.

Dr. W. C. KRAUSS reported that the committee on publishing proceedings of the Academy in the BUFFALO MEDICAL JOURNAL could not make a report, as the editor was out of town, and asked for further time. On motion the committee was given until the next quarterly meeting (September) to report.

A letter was read from Dr. Charles R. Dickson, president of the American Electro-therapeutic Association, extending a cordial invitation to the members to attend the eighth annual meeting of the association, which will be held in Buffalo, September 13-15, 1898, also asking that a delegate be sent from the academy. Dr. M. D. Mann moved that the invitation be accepted and the chair appoint a delegate. Carried.

Dr. Floyd S. Crego was appointed as such delegate.

Notice of the following proposed amendments to the by-laws, recommended by the council, was read by the secretary :

"That names of members who are six months or more in arrears for dues and the amounts due be posted in a conspicuous place in the academy rooms until same are paid."

"That Article XIII., section 3, of the by-laws be amended to read, any fellow failing to pay his dues for two years shall forfeit his fellowship."

The section on obstetrics provided the following program : Discussion and diagnosis of suppurative forms of intrapelvic disease and surgical management, by Dr. Joseph Price, Philadelphia, Pa. Discussion followed in which Drs. Mann, Hayd, Crockett and Benedict participated. The attendance at the meeting was fifty-six.

Adjourned at 10 p. m.

